# Paternal facilitation of maternal role usurpation and mother–child bond rupture in stepfamilies: a proposal for early detection and protective measures

**DOI:** 10.3389/fpsyg.2026.1959681

**Published:** 2026-10-28

**Authors:** Ana Belén Fernández

**Affiliations:** Anesthesiology and Postsurgical Intensive Care Department, Ntra Sra De Candelaria University Hospital, Santa Cruz de Tenerife, Spain

**Keywords:** ambiguous loss, maternal role usurpation, mother–child bond, parental repartnering, parenting plans, paternal facilitation, stepfamily formation, vicarious violence

## Abstract

Divorced parents frequently begin new relationships during the early stages of family reorganization, and their new partners may become involved with the children before any formal stepfamily exists. Yet family law barely regulates the role of these new partners. This Perspective focuses on one specific configuration: a father who repartners under shared custody, and a mother who is not informed of the new partner’s involvement with children under 12. I describe how, with the father’s active or passive facilitation, the new partner progressively assumes maternal functions, to the point of usurping the maternal role and breaking the mother–child bond. Children, as active participants whose responses vary with age, may become caught in loyalty conflict and in a grief for their mother that they do not recognize as such, sustained by a rescue narrative that presents the new partner as the one who gave them the affection their mother could not. For the mother, the loss is ambiguous, lacks social recognition and may lead to a state of shock and paralysis and to severe cumulative trauma, with symptoms compatible with complex post-traumatic stress disorder, that is difficult to resolve and has devastating consequences for her health, including suicide risk. I propose a preventive circuit linked to the divorce agreement: a universal, judicially enforceable clause requiring disclosure of the new partner’s involvement and definition of that partner’s role, and risk screening at separation that triggers mandatory psychoeducation, mediation and monitoring in at-risk families. The circuit may also contribute to the early detection of vicarious violence, and its effectiveness depends on professionals trained in these dynamics.

## Introduction

Family law now regulates the relationship between separated parents through parenting plans, custody schedules and decision-making rules. What remains largely unregulated is the role of a parent’s new partner, who may come to occupy a substantial caregiving role without any formal limit on their conduct or authority, whether or not the couple cohabits. Many parents manage these transitions carefully (Kang et al., 2023; Kang and Nielson, 2025). My concern is narrower: situations in which a new partner becomes regularly involved with minor children while the other parent is not informed, with the risk that, given the father’s passivity, the maternal role is displaced or usurped.

To avoid generalizing across diverse stepfamilies (Ganong and Coleman, 2017), I focus on one configuration: a *father* who repartners after separation; children who live with him for substantial periods under shared custody; children under 12 years of age; and, as the relationship at risk, that between the *mother* — the parent who has not repartnered — and her children. Similar processes may occur in other configurations, but I do not assume they unfold in the same way. I use the term *new partner* rather than “third party” to distinguish this figure from grandparents, half-siblings or other relatives.

I call the outcome of concern *usurpation of the maternal role*: the gradual, cumulative assumption by a new partner of the caregiving, decision-making and relational functions that define a child’s relationship with their mother, accompanied by the progressive weakening of the affective mother–child bond, which at its extreme breaks down entirely. I use “usurpation” to name the severity of this outcome, not to attribute intent.

This Perspective draws on my own experience. I regard it as a legitimate source of knowledge: the phenomenon described often goes unnoticed by those without specialized training, and first-hand knowledge makes it possible to identify and name it (Ellis et al., 2011). At the same time, I distinguish its role from that of evidence: this lived knowledge has shaped the questions I ask, whereas the claims of the article rest on published literature, are framed as testable propositions and are weighed against alternative explanations.

## Conceptual boundaries

Usurpation of the maternal role is related to three neighboring constructs, from which it differs. Parental gatekeeping describes a parent’s attitudes and behaviors that facilitate or restrict the other parent’s relationship with the child (Austin et al., 2013); usurpation concerns not access but the substitution of a role, and its principal agent is another adult, facilitated by the parent. Parental alienation, a contested construct, describes a child’s unjustified rejection of a parent; usurpation may occur without explicit rejection, is defined by the observable conduct of adults and requires no diagnosis of the child or of either parent. Loyalty conflict is the child’s inner experience of being caught between attachment figures; in my model it is a possible consequence of usurpation, not its definition.

I operationalize usurpation through three observable components: (1) *functional substitution*, when the new partner assumes parental functions that belong to the parents (Table 1); (2) *informational exclusion*, when the mother is not informed of the new partner’s involvement; and (3) *symbolic displacement*, when the new partner is presented, or presents herself, as a maternal figure, or the mother is disqualified in front of the children. It is the joint and sustained presence of the three components, not an isolated event, that defines the process, and each can be assessed through the indicators in Tables 1, 2.

**Table 1:** Facilitating behaviors: parental functions assumed by a new partner, why they belong to the parents, and the corresponding safeguard.

Conduct	Why it belongs to the parents	Safeguard
Collecting the child from school	Reunion with an attachment figure; public act before the school community	Reserved to the parents^a^
Attending medical appointments or making health decisions	Decisions within joint parental authority; access to the child’s clinical information	Reserved to the parents^a^
Deciding to buy the child a mobile phone	Decision on the child’s digital access, within joint parental authority	Reserved to the parents^a^
Exercising parental control over the child’s devices	Supervision of the child’s digital life belongs to the parents; control held by the new partner excludes the mother	Parental control held by the parents, with no access by the new partner
Direct, regular communication with the child (e.g., daily messages)	Creates a private channel outside one parent’s knowledge and intensifies loyalty conflict	Only with both parents’ agreement
Providing bank cards or opening accounts	Legal representation and finances of the minor (Art. 162, Civil Code)	Reserved to the parents
Presenting herself as a maternal figure or endorsing comparisons with the mother	Sustains the rescue narrative and displaces the mother	Activation indicator^b^

a Universal clause: except in the prolonged absence or incapacity of a parent or in an emergency, and always with the other parent’s knowledge.

b Triggers reassessment and, in moderate- or high-risk families, review of the arrangements.

**Table 2:** Proposed risk levels for displacement of the maternal bond, indicators at separation, and responses.

Risk level	Indicators at separation	Response
Low	Cooperative coparenting; information shared between households; no relevant conflict	Universal clause only^a^
Moderate	Unresolved resentment or anger; high interparental conflict; breakdown of communication between households; young children^b^ combined with any of the above	Psychoeducation^c^; early mediation; scheduled review; reassessment when either parent repartners^d^
High	Moderate-level indicators plus prior restrictive gatekeeping, denigration of the other parent in front of the children, or a history of noncompliance with agreements	As for moderate, plus monitoring by the psychosocial team and judicial review at defined intervals

Indicators are proposed for empirical validation and do not constitute a validated instrument.

a Disclosure to the other parent of a new partner’s regular involvement. The new partner may provide everyday care but does not assume parental functions or the maternal role with children under 12, except in the prolonged absence or incapacity of a parent or in an emergency, and always with the other parent’s knowledge.

b Under 12 years of age.

c Mandatory for both parents, on loyalty conflict, children’s developmental needs and communication about new relationships (Kang and Nielson, 2025).

d Activation indicators: concealment of the new partner, assumption of parental functions without agreement, denigration of the mother in front of the children, and emerging withdrawal of the children from her.

## The courtship stage

Stepfamily formation involves several transitions, typically beginning with a parent’s dating and potentially progressing to cohabitation and remarriage (Anderson and Greene, 2005; Ganong and Coleman, 2017). My argument concerns the courtship stage and the early transition into shared household life, when the new partner’s role is least defined. Disclosure of a new relationship to children and to the co-parent is an active decision that varies with parents’ perceptions (Kang et al., 2023); stepchildren construct their own interpretations of it (Kang and Zhou, 2025), and how it is communicated can protect or strain the parent–child bond (Kang and Nielson, 2025). The effect of non-disclosure to the other parent on that parent’s relationship with the child has not been examined.

Children are not passive recipients of adults’ decisions. Stepchildren respond to, reciprocate or reject a stepparent’s affinity-seeking (Ganong et al., 1999), and their reactions range from acceptance to resistance and ambivalence, changing over time (Ganong and Coleman, 2017). Affinity-seeking is not in itself harmful. The concern is affinity-seeking that occurs behind the other parent’s back and without agreed limits. The child then builds a relationship with the new partner, as the literature predicts, within a relational field the mother cannot see. Affectionate gestures within a concealed relationship may create an unspoken sense of obligation — the invisible loyalty described by Boszormenyi-Nagy and Spark (1973).

A new partner’s involvement can also be a resource for the child. A meta-analysis of 56 studies found that high-quality stepparent–child relationships are associated with greater psychological and academic well-being and fewer psychological and behavioral problems, with no significant differences by stepparent gender (Jensen, 2022). Close ties to the resident parent, the nonresident parent and the stepparent are each associated with better outcomes (King, 2006), and the quality of the prior parent–child relationship fosters a good relationship with the new partner (King, 2009). This evidence is consistent with my argument: the benefit arises when the new partner adds to existing bonds, not when the new partner replaces them.

The literature describes a healthy stepmother role as “a mothering but not a mother role” (Weaver and Coleman, 2005): caring for the children without occupying the mother’s place. Other stepmothers adopt a “nuclear” model in which they want the children to call them “mom,” some wish to be taken for the biological mother, they limit contact with the former partner and they emphasize their role as primary mother (Church, 1999). It is in this second case that boundaries are crossed. In stepfamilies, the name given to each adult can express a struggle over each person’s position in the family (Furstenberg and Cherlin, 1991). When a young child asks the father’s new partner “are you my mum?”, the question reflects not only immaturity but the confusion produced by boundaries the adults have not defined. When this is accompanied, explicitly or implicitly, by the message that the mother is not valid or is bad, the secure attachment to her is itself at risk. Children build internal working models of their attachment figures (Bowlby, 1969), and a repeated narrative that disqualifies the mother may distort that representation and weaken a previously secure bond, consistent with the association between such experiences and insecure attachment in adulthood (Baker and Ben-Ami, 2011).

Developmental stage shapes this process. Preschool children (approximately 3–5 years) depend on continuity with primary attachment figures (Bowlby, 1969), have a concrete understanding of family roles and can report little of what happens in the other household. In middle childhood (6–11), children recognize competing loyalties and are more likely to feel caught, but can also express preferences and resist a new partner’s role. In preadolescence (approximately 10–12 years), the child may adopt the narrative received as their own and express a wish to change residence, so that refusing to see the mother appears to be an autonomous decision when it may be the outcome of the process described here. Adolescents evaluate competing narratives more independently. My argument applies with most force to the preschool and early school years.

## The role of the repartnering father

In separations where pain, resentment or anger remain unresolved, the parent who begins a new relationship controls information and sets, or fails to set, the limits of the new partner’s role. When the father does not inform the mother, does not define that role, or presents the new partner as a more adequate maternal figure, he facilitates the mother’s displacement, even if not consciously. Facilitation can be active — delegating maternal functions or endorsing unfavorable comparisons — or passive: failing to correct such comparisons or to intervene when the new partner oversteps, and allowing the mother’s image to disappear from the children’s daily lives while another woman occupies her place.

Table 1 summarizes common forms of this facilitation, such as the new partner collecting a young child from school — a public moment of reunion with particular emotional weight in early childhood (Ainsworth et al., 1978) — attending medical appointments, deciding to buy the child a mobile phone or exercising parental control over the child’s devices. Under Spanish law, parental authority is exercised jointly (Art. 156, Civil Code) and legal representation of minors belongs to the parents (Art. 162); a new partner holds neither. Such conduct undermines the joint exercise of parental authority and can cause serious harm, setting in motion loyalty conflict and the gradual tearing of the child away from the mother.

Beyond any legal requirement, the repartnering parent has a moral obligation, grounded in respect for the children, to inform the other parent before bringing a new partner into the children’s intimate life and to define that relationship clearly, especially when the children are young. The safeguards proposed below give legal form to that obligation.

## Consequences for the mother

Non-disclosure of a new partner’s involvement functions as an indirect form of restrictive gatekeeping (Austin et al., 2013): it does not restrict the mother’s access but deprives her of the information she needs, which makes it harder to detect.

For the mother, the result resembles ambiguous loss: a relationship that remains partly present and can be neither retained nor relinquished (Boss, 1999, 2006). Because no legal or social category names it, it is also disenfranchised grief (Doka, 1989). A double bind compounds it (Bateson et al., 1956): setting boundaries may be read as rigidity, expressing distress as instability and, perversely, trying to protect her children as vindictiveness (Spearman et al., 2023). This double bind is reproduced in the judicial sphere, where it can become secondary victimization, a form of institutional violence: even the mother’s withdrawal to protect herself is used against her as evidence of indifference or abandonment. When legal professionals are unaware of this dynamic, the proceedings themselves can leave her with no way out. The United Nations Special Rapporteur on violence against women and girls has documented how, in custody proceedings, mothers’ actions are frequently interpreted against them (Alsalem, 2023). Repeated unsuccessful efforts may lead to self-attributed helplessness (Abramson et al., 1978), and the mother may withdraw from everyday parenting or relinquish residential time when children show distress at transitions. Non-resident mothers experience their situation as a threat to identity and work to resist the “bad mother” label (Kielty, 2008a, 2008b); some come to internalize it (Tangir et al., 2017).

The harm may be compounded by what I call a *rescue narrative*: the portrayal of the new partner as the one who gave the children the affection their mother, presented as unstable or inadequate, could not give them. When this narrative is directed at the mother herself — the new partner claiming that she is the one who has had to raise her children — it *usurps the upbringing itself*, rewriting the mother’s history with her children and denying her even the fact of having been a mother.

In its severe forms, this experience is traumatic, and its impact accumulates with each loss and each failed attempt to restore the bond. Chronic interpersonal stress leaves a documented neurobiological imprint (McEwen, 1998), and complex trauma is not exclusive to childhood: it is also associated with prolonged interpersonal trauma in adulthood (Palic et al., 2016). I therefore propose, as a clinical hypothesis to be evaluated, that in the most severe cases these mothers may present symptoms compatible with complex post-traumatic stress disorder as defined in ICD-11 (World Health Organization, 2019). The interpersonal theory of suicide identifies thwarted belongingness and perceived burdensomeness as central contributors to suicidal desire (Joiner, 2005; Van Orden et al., 2010); a mother who loses her place in her children’s lives and is told that she caused that loss may be exposed to both. Parents reporting marginalization after separation show elevated depression (Verrocchio et al., 2019), although such studies rely on self-selected samples and on the contested construct of parental alienation, whose judicial use is discouraged in Spain (Boletín Oficial del Estado, 2021, Art. 11.3); my proposal does not depend on that construct. No study has yet examined suicide risk in this specific situation; in a small sample, also self-referred, of parents who considered themselves separated from their children, 23% reported a previous suicide attempt (Lee-Maturana et al., 2020). The scarcity of research is consistent with the invisibility of the phenomenon rather than evidence of its absence. Meanwhile, professionals should routinely assess depression, hopelessness and suicidal ideation in displaced mothers.

## The children’s grief

For children, usurpation of the maternal role constitutes a serious assault on their psychological integrity. Spanish Organic Law 8/2021 defines violence against children as any act, omission or negligent treatment that deprives minors of their rights and well-being, or that threatens or interferes with their physical, psychological or social development (Boletín Oficial del Estado, 2021, Art. 1.2), and the Convention on the Rights of the Child requires States to protect children from all forms of mental violence (United Nations, 1989, Art. 19). Depriving a child of the maternal bond without justification may fall within that definition. Boss (1999) described ambiguous loss in children of divorce; here it has an additional feature: children move away from their mother without knowing the real reason, often through the rescue narrative, so their grief is hidden even from themselves. In childhood, cumulative trauma is associated with greater symptom complexity (Cloitre et al., 2009). Children turned against a parent lose that parent’s support and may later feel guilt and shame (Amato and Afifi, 2006). Years later, retrospective studies describe lower self-esteem, depression, insecure attachment and difficulty trusting others (Baker, 2005; Baker and Ben-Ami, 2011), and exposure to loyalty-conflict behaviors is associated with later psychological distress (Verrocchio et al., 2015). The rupture need not be permanent: around 81% of adult children estranged from their mother later reconciled with her (Reczek et al., 2023), although it concerns estrangement that began in adulthood. The rescue narrative may be questioned in adolescence or adulthood, opening a possibility of reunion, but also of guilt.

## The mother as witness to her children’s grief

One dimension of the harm is barely addressed in the literature: the mother does not only lose her children, she witnesses their grief. Young children experience the rupture as a real loss at a time when they can neither understand nor name it, and their confusion is expressed in crying, distress at transitions or questions they cannot formulate. The mother, the very figure they would seek for comfort, finds herself removed from her role and unable to intervene. Over time, the confusion may become such that the child cries when the mother comes to collect them, or even does not want her to visit: for her this is especially painful and easily read as rejection, although it more likely reflects loyalty conflict. That distress may in turn be used by the father as proof that the mother is harmful and must be removed from the children’s lives permanently, closing the double bind: the suffering caused by displacement becomes the argument for completing it. Research on post-separation abuse describes how children’s reactions are used in family proceedings to restrict contact with the mother, and how mothers who witness that suffering experience psychological anguish and declining health, linked to retraumatisation and the helplessness of being unable to console them (Millar et al., 2026).

Displacement is not inevitable. Cooperative coparenting, warm parenting by both parents, residential arrangements, the pace of repartnering, the quality of the pre-existing bond and children’s characteristics all shape outcomes (Ganong and Coleman, 2017). Most co-parents and new partners do not intend harm; unresolved post-separation affect can surface through silence, permissiveness or subtle undermining without the actors recognizing it. The appropriate response is therefore preventive and structural: safeguards should specify observable conduct rather than judge motivation.

## A preventive legal–clinical circuit

### Guiding principle

Adult conduct during this stage should be guided by the protection of the children and their bond with both parents, as already required by the best interests of the child (Boletín Oficial del Estado, 1996, Art. 2) and the child’s right to maintain regular contact with both parents (United Nations, 1989, Art. 9.3).

Spanish family law regulates co-parenting through the *convenio regulador* (Art. 90, Civil Code) and, in some regions, mandatory parenting plans, but neither requires disclosure of a new partner’s involvement. Case law treats a new partner’s circumstances as relevant only reactively, once risk or a change of circumstances has been demonstrated (STS 1881/2025, STS 1166/2024, 2024). I propose a two-level circuit linked to the divorce agreement, applicable to any repartnering parent regardless of gender (Tables 1, 2).

#### Universal level

Every agreement involving minor children would include a standard clause requiring timely notification when a new partner becomes a regular presence in the children’s lives. The clause would distinguish between the everyday care a new partner may provide in the father’s home, parental functions (decision-making, legal representation, and dealings with school or health services) and the maternal role. With children under 12, the new partner may not assume parental functions or occupy the maternal role, except in the prolonged absence or incapacity of a parent or in an emergency, and always with the other parent’s knowledge; nor may the new partner initiate direct, regular communication with the child without both parents’ agreement. It would not prohibit a warm relationship; it would place it within “a mothering but not a mother role” (Weaver and Coleman, 2005). Because the new partner is not a party to the agreement, the clause binds the parent, and it would be judicially enforceable by the other parent through the mechanisms for enforcing family measures (Art. 776, Civil Procedure Act).

#### Risk-based level

Screening when the agreement is drafted would identify families at risk, regardless of whether a new partner already exists, since unresolved anger and high conflict precede any new partner. In moderate- and high-risk families, psychoeducation would be mandatory for both parents as a child-protection measure (Art. 158, Civil Code); refusal would be taken into account in custody decisions and would place the family at high risk.

Displacement lies on a continuum. At one end it results from non-conscious systemic drift; at the other, a father may deliberately use the new partner and the children to cause suffering to the mother, which could fall within the definition of vicarious violence in the Spanish draft law currently before Parliament (Congreso de los Diputados, 2026). This end of the continuum is consistent with research on post-separation abuse, which encompasses psychological, legal and economic abuse and the weaponising of children by a former partner (Spearman et al., 2023, 2024), and which is fostered by gendered norms of caregiving (Davies et al., 2009) and by stereotypes that portray mothers as obstructive or vindictive (Spearman et al., 2023). This use of children as an instrument of control may intensify after separation, when the abuser has less direct access to the mother (Hardesty et al., 2024). In a qualitative study with mothers supported by Women’s Aid, including mothers whose children lived with their fathers, participants described fathers undermining their maternal authority and seeking to erase them from their children’s lives, and in some cases new partners also mistreated the children; the authors adopt the term “child and mother sabotage” as an alternative to parental alienation (Millar et al., 2026). Because the circuit does not require intent to be proven, it can help prevent, detect early and mitigate some such situations. Its effectiveness depends on judges, prosecutors, psychosocial teams, mediators and family lawyers trained in contemporary stepfamily dynamics and in vicarious violence, which is particularly perverse because it turns the children’s bond with their mother into the instrument used to harm her. Such training should include the hearing of children: their views must be taken into account and respected (United Nations, 1989, Art. 12), and professionals should be able to assess whether what a child expresses reflects their own experience or a narrative received, particularly once those views acquire legal weight; in Spain, a child is in any case considered mature enough to be heard from the age of 12 (Boletín Oficial del Estado, 1996, Art. 9).

## Discussion

Non-disclosure during the courtship stage, a new partner’s unbounded involvement and the father’s facilitation together create a structural vulnerability with serious consequences for children and their mothers. This Perspective addresses one family configuration; its mechanisms derive from theory and adjacent evidence, and outcomes depend on the moderating factors described. This is a serious problem: the new partner’s conduct and the father’s permissiveness, whether conscious or not, constitute a symbolic killing of the maternal figure, leaving children whom the law is bound to protect without a mother in practice, even though their mother is alive. In its extreme forms, it may also cost mothers their lives, given the suicide risk described. Despite its gravity, this conduct currently has no legal consequences unless intent to harm is established. It is precisely for this reason that I propose to regulate it. Future research should examine how often co-parents are informed of a new partner’s involvement and when; whether concealment predicts changes in the mother–child relationship; children’s own accounts across developmental stages; how many children later resume their relationship with their mother; and the effects of disclosure clauses where they exist. If this phenomenon is neither recognized nor addressed, there is reason to fear that the number of children torn away from their mothers will grow. Clinical psychology and family law are well placed to act together, protecting children from chronic divided loyalty and mothers from an unrecognized loss. It is, therefore, a pressing current concern for both disciplines: as recently stated in *The Lancet*, post-separation abuse is “an ignored public health crisis and preventable injustice” (Prah and Gostin, 2025).

## Data Availability

The original contributions presented in the study are included in the article/supplementary material, further inquiries can be directed to the corresponding author.
